# Assessment of Cystatin C Level for Risk Stratification in Adults With Chronic Kidney Disease

**DOI:** 10.1001/jamanetworkopen.2022.38300

**Published:** 2022-10-25

**Authors:** Jennifer S. Lees, Elaine Rutherford, Kathryn I. Stevens, Debbie C. Chen, Rebecca Scherzer, Michelle M. Estrella, Michael K. Sullivan, Natalie Ebert, Patrick B. Mark, Michael G. Shlipak

**Affiliations:** 1 School of Cardiovascular and Metabolic Health, University of Glasgow, Glasgow, United Kingdom; 2 Glasgow Renal and Transplant Unit, Queen Elizabeth University Hospital, Glasgow, United Kingdom; 3 Renal Unit, Mountainhall Treatment Centre, NHS Dumfries and Galloway, Dumfries, United Kingdom; 4 Kidney Health Research Collaborative, Department of Medicine, University of California San Francisco and San Francisco VA Health Care System, San Francisco; 5 Genentech/Roche, South San Francisco, California; 6 Institute of Public Health, Charité University Hospital, Berlin, Germany

## Abstract

**Importance:**

Kidney function is usually estimated from serum creatinine level, whereas an alternative glomerular filtration marker (cystatin C level) associates more closely with future risk of cardiovascular disease (CVD) and mortality.

**Objectives:**

To evaluate whether testing concordance between estimated glomerular filtration rates based on cystatin C (eGFRcys) and creatinine (eGFRcr) levels would improve risk stratification for future outcomes and whether estimations differ by age.

**Design, Setting, and Participants:**

A prospective population-based cohort study (UK Biobank), with participants recruited between 2006-2010 with median follow-up of 11.5 (IQR, 10.8-12.2) years; data were collected until August 31, 2020. Participants had eGFRcr greater than or equal to 45 mL/min/1.73 m^2^, albuminuria (albumin <30 mg/g), and no preexisting CVD or kidney failure.

**Exposures:**

Chronic kidney disease status was categorized by concordance between eGFRcr and eGFRcys across the threshold for hronic kidney disease (CKD) diagnosis (60 mL/min/1.73 m^2^).

**Main Outcomes and Measures:**

Ten-year probabilities of CVD, mortality, and kidney failure were assessed according to CKD status. Multivariable-adjusted Cox proportional hazards models tested associations between CVD and mortality. Area under the receiving operating curve tested discrimination of eGFRcr and eGFRcys for CVD and mortality. The Net Reclassification Index assessed the usefulness of eGFRcr and eGFRcys for CVD risk stratification. Analyses were stratified by older (age 65-73 years) and younger (age <65 years) age.

**Results:**

There were 428 402 participants: median age was 57 (IQR, 50-63) years and 237 173 (55.4%) were women. Among 76 629 older participants, there were 9335 deaths and 5205 CVD events. Among 351 773 younger participants, there were 14 776 deaths and 9328 CVD events. The 10-year probability of kidney failure was less than 0.1%. Regardless of the eGFRcr, the 10-year probabilities of CVD and mortality were low when eGFRcys was greater than or equal to 60 mL/min/1.73 m^2^; conversely, with eGFRcys less than 60 mL/min/1.73 m^2^, 10-year risks were nearly doubled in older adults and more than doubled in younger adults. Use of eGFRcys better discriminated CVD and mortality risk than eGFRcr. Across a 7.5% 10-year risk threshold for CVD, eGFRcys improved case Net Reclassification Index by 0.7% (95% CI, 0.6%-0.8%) in older people and 0.7% (95% CI, 0.7%-0.8%) in younger people; eGFRcr did not add to CVD risk estimation.

**Conclusions and Relevance:**

The findings of this study suggest that eGFRcr 45 to 59 mL/min/1.73 m^2^ includes a proportion of individuals at low risk and fails to capture a substantial proportion of individuals at high-risk for CVD and mortality. The eGFRcys appears to be more sensitive and specific for CVD and mortality risks in mild CKD.

## Key Points

**Question:**

Does estimation of kidney function by cystatin C level improve risk-stratification for cardiovascular disease and mortality?

**Findings:**

In this cohort study, patients with estimated glomerular filtration rate based on serum creatinine level less than 60 mL/min/1.73 m^2^ were at low or high risk of future cardiovascular disease and death. Estimated glomerular filtration rate based on cystatin C acurately stratified risk in these individuals.

**Meaning:**

The findings of this study suggest that, in the absence of cystatin C testing, estimated glomerular filtration rate inadequately distinguishes the broader risks associated with mild chronic kidney disease based on serum creatinine level.

## Teaser

This cohort study compares the use of cystatin C vs creatinine level for detection of high-risk chronic kidney disease.

## Introduction

Kidney Disease Improving Global Outcomes guidelines recommended estimation of glomerular filtration rate based on cystatin C level (eGFRcys) testing for confirmation of chronic kidney disease (CKD) in the absence of other markers of kidney damage and in circumstances when estimated glomerular filtration rate is based on serum creatinine (eGFRcr) level may be less accurate.^1^ Glomerular filtration rate decreases with age^2^; however, muscle mass and activity are the main determinants of serum creatinine levels at fixed GFR.^3^ Older people tend to have lower muscle mass,^4^ leading to lower creatinine generation, which may result in overestimation of GFR.^3^ These competing factors lead eGFRcr to have potential discordance with eGFRcys, which may be especially pronounced in older adults. Furthermore, eGFRcys has a greater association vs eGFRcr with future risk of cardiovascular disease (CVD) and death.^5,6^ However, the recommendation to conduct concordance testing for CKD with cystatin C is rarely implemented.^7^

To examine whether the use of cystatin C as a concordance test for CKD could improve detection of high-risk CKD, we compared the estimated prevalence of CKD based on eGFRcr, eGFRcys, and parallel analysis (eGFRcr-cys) among people with an eGFRcr greater than or equal to 45 mL/min/1.73 m^2^ and no albuminuria. We stratified the analyses by age to assess whether concordance testing had similar value in younger compared with older adults. We hypothesized that there would be substantially higher risk for adverse outcomes (including CVD, all-cause mortality, and kidney failure) in the subset of patients with CKD confirmed by eGFRcys testing.

## Methods

### Participants and Baseline Data Collection

Participants were from the prospective cohort study UK Biobank. Details of the recruitment procedure and protocol have been described previously^8,9^ (eMethods 1 in the Supplement). Of 502 536 participants aged 37 to 73 years initially recruited, we excluded 76 who withdrew ongoing consent for follow-up; 33 491 participants with missing biochemistry data at baseline; 24 006 with urine albumin to creatinine ratio greater than or equal to 30 mg/g, because this level signifies CKD regardless of eGFR; 15 998 with a history of myocardial infarction, stroke, or kidney failure; and 563 with eGFRcr less than 45 mL/min/1.73 m^2^ at baseline, leaving 428 402 participants in the final cohort. Participants provided written informed consent for baseline phenotyping and prospective follow-up, with electronic linkage to hospital health records and death registries, allowing extraction of diagnoses from these records using *International Statistical Classification of Disease, Tenth Revision* (*ICD-10*) codes. No financial compensation was provided. Ethical approval was granted by the North West Multi-Centre Research Ethics Committee. This study is reported according to the Strengthening the Reporting of Observational Studies in Epidemiology (STROBE) reporting guideline.

The procedure of biochemical sampling for UK Biobank has been described previously^10,11,12^ (eMethods 1 in the Supplement). For the main analyses, eGFR was calculated according to Chronic Kidney Disease Epidemiology Collaboration (CKD-EPI) equations without race coefficients^13^ from eGFRcr levels, eGFRcys levels, and the combined equation with creatinine and eGFRcr-cys. Urine albumin to creatinine ratio was calculated to define albuminuria.

Reduced eGFR was first defined according to the Kidney Disease Improving Global Outcomes classification system^1^ and according to whether eGFRcr was concordant with eGFRcys, because eGFRcys is more likely to be discordant with eGFRcr, as it does not incorporate serum creatinine levels (unlike eGFRcr-cys), and eGFRcys has been shown to be the GFR estimate demonstrating greater association with CVD and all-cause mortality across the spectrum of eGFR.^5,6^

After excluding participants with eGFRcr less than 45 mL/min/1.73 m^2^ (as per study eligibility criteria), CKD status was defined as follows (G indicates that staging was made using only glomerular filtration criteria and not albuminuria): (1) no CKD (eGFRcr ≥60 mL/min/1.73 m^2^ and eGFRcys ≥60 mL/min/1.73 m^2^); (2) eGFRcr G3 (eGFRcr <60 mL/min/1.73 m^2^ and eGFRcys ≥60 mL/min/1.73 m^2^; (3) eGFRcys G3 (eGFRcr ≥60 mL/min/1.73 m^2^ and eGFRcys <60 mL/min/1.73 m^2^); and (4) both G3 (eGFRcr <60 mL/min/1.73 m^2^ and eGFRcys <60 mL/min/1.73 m^2^).

We conducted parallel analyses, defining CKD status using eGFRcr-cys rather than eGFRcys as the concordance test, because this combined equation has repeatedly been shown to approximate measured GFR more closely than either eGFRcr or eGFRcys alone.^13,14,15^ In sensitivity analyses, we defined CKD status using the 2009 eGFRcr by the CKD-EPI equation including race coefficients (2009 eGFRcr),^14^ because this would have been the recommended measure to diagnose CKD at the point of sampling.

Baseline variables of interest were selected according to atherosclerotic risk factors included in risk prediction tools for CVD^16^ and include age; sex; smoking status; systolic and diastolic blood pressure; total, high-density lipoprotein, and low-density lipoprotein cholesterol levels; self-reported history of diabetes or hypertension; and use of blood pressure– or cholesterol-lowering medications. Baseline medical conditions, medications, and smoking history were self-reported via a nurse-led interview.

### Outcomes

There were 3 main outcomes of interest: fatal or nonfatal CVD, all-cause mortality, and kidney failure. Fatal or nonfatal CVD was defined as the first event in the follow-up period of myocardial infarction, stroke (ischemic and hemorrhagic), or cardiovascular death, censored for non-CVD causes. Myocardial infarction and stroke were defined by UK Biobank outcome definitions.^17^ Cardiovascular death was defined by death registration–reported death from a cardiovascular cause according to *ICD-10* codes (I00-I99). All-cause mortality was defined from the date of death from linked death certification records according to a prespecified algorithm.^17^ Because follow-up biochemical data were not routinely available, kidney failure was defined from hospital admission data according to a prespecified algorithm, using *ICD-10* and Office of Population Censuses and Surveys Classification of Interventions and Procedures, Version 4 codes to identify participants who required maintenance kidney replacement therapy or CKD stage 5 (eMethods 2 in the Supplement).^18^ Participants were followed up from enrollment in the UK Biobank until the first occurrence of an outcome of interest, death, or the end of data collection (August 31, 2020).

### Statistical Analysis

Baseline data were described across CKD categories after confirmatory testing by eGFRcys and eGFRcr-cys. Continuous variables are displayed as mean (SD) and nonnormal data are displayed as median (IQR). Nonnormality was confirmed by visual inspection of histograms and quantile-quantile plots. Categorical risk factors are displayed as proportions (percent).

Missing data were multiply imputed by chained equations, using the average of 5 separately imputed data sets. The proportion of missing data for each imputed variable was less than 9% (maximum 8.5% for high-density lipoprotein cholesterol but 0.2% for low-density lipoprotein and total cholesterol; 3% for urine albumin to creatinine ratio), and the data were assumed to be missing at random.

Quantile regression was used to estimate median (IQR) change in eGFR with increasing age across age brackets (age <45, 45 to <50, 50 to <55, 55 to <60, 60 to <65, 65 to <70, and 70-73 years) as a measure of discordance among the eGFR measures. Median levels of eGFR by method (eGFRcr vs eGFRcr-cys vs eGFRcys) were compared within each age bracket using the Wilcoxon signed rank test. A scatterplot was used to illustrate the concordance between eGFRcr and eGFRcys, with locally weighted linear regression trend lines plotted for each age bracket.

Across the full cohort, 10-year absolute risks of the outcomes of interest were assessed using the cumulative incidence function, stratified by CKD status. We stratified analyses by age as older (65-73 years) or younger (<65 years). The age threshold was selected based on the recommendations for the age-adapted criteria for CKD diagnosis.^19^

To assess the association between each eGFR measure and CVD, penalized splines of eGFR against hazard ratios of CVD were plotted, adjusted for age; sex; smoking status; systolic and diastolic blood pressure, total, high-density lipoprotein, and low-density lipoprotein cholesterol; history of diabetes or hypertension; and use of blood pressure– or cholesterol-lowering medications, and albuminuria. Splines were stratified by age in older and younger participants. An eGFR of 90 mL/min/1.73 m^2^ was considered the reference value.

Cox proportional hazards regression models were constructed to assess the risk of outcomes of interest by CKD category, adjusting for atherosclerotic risk factors as above, and censoring for all-cause mortality. The proportional hazards assumption was tested by plotting Schoenfeld residuals and no violation was found. Using area under receiver operating curves, we assessed the individual discriminative abilities of eGFRcys, eGFRcr, and selected traditional atherosclerotic risk factors (age, systolic blood pressure, and low-density lipoprotein cholesterol, as these are expressed as continuous variables) for fatal and nonfatal CVD and all-cause mortality in older and younger participants. Area under receiver operating curves then assessed incremental discrimination of eGFRcr and eGFRcys for CVD and mortality over a base model of traditional cardiovascular risk factors (eMethods 3 in the Supplement). The Net Reclassification Index (NRI) was used to assess the predictive utility of adding eGFRcys to a base model of cardiovascular risk factors to assess risk across a 7.5% cutoff point for 10-year risk of CVD—the threshold that would warrant consideration of a moderate- or high-intensity statin.^16^ The 95% CIs reported were obtained from 1000 bootstrap replicates.

Statistical analyses were conducted in R for Statistical Software in RStudio, version 1.4.1106 (R Foundation for Statistical Analysis) for Mac using tidyverse, tableone, survival, survminer, nephro, mice, quantreg, cmprsk, pROC, and nricens packages.

## Results

There were 428 402 participants with median age of 57 (IQR, 50-63) years, 237 173 (55.4%) were women, and 191 229 (44.6%) were men. Among 76 629 older participants (median follow-up, 11.4; IQR, 10.6-12.1 years), there were 9335 deaths, 5205 CVD events (2158 CVD-associated deaths), and 46 kidney failure events. Among 351 773 younger participants (median follow-up, 11.6; IQR, 10.9-12.3 years), there were 14 776 deaths, 9328 CVD events (3117 CVD-associated deaths), and 124 kidney failure events.

### Baseline Characteristics

Compared with individuals without evidence of CKD by any criterion, those with evidence of CKD G3 by both eGFRcr and eGFRcys were more likely to have baseline diabetes and hypertension and to use blood pressure– and cholesterol-lowering medications. Similarly, participants with evidence of CKD G3 by eGFRcys (Table) and eGFRcr-cys (eTable 1 in the Supplement) had a higher prevalence of baseline comorbidities and medication use compared with those with CKD G3 by eGFRcr only.

**Table.** Baseline Characteristics of Participants by CKD Status in Concordance Testing Between eGFRcr and eGFRcys^a^

Baseline characteristics	No. (%)							
	Older participants (age 65-73 y)				Younger participants (age <65 y)			
	No CKD	eGFRcr G3	eGFRcys G3	Both G3	No CKD	eGFRcr G3	eGFRcys G3	Both G3
No.	68 335	737	6278	1279	342 614	1294	6887	978
Age, median (IQR), y	67 (66-68)	67 (66-68)	67 (66-68)	67 (66-69)	55 (48-60)	60 (55-62)	61 (57-63)	61 (58-63)
Sex								
Male	32 652 (48)	266 (36)	2651 (42)	553 (43)	151 707 (44)	398 (31)	2629 (38)	373 (38)
Female	35 683 (52)	471 (64)	3627 (58)	726 (57)	190 907 (56)	896 (69)	4258 (62)	605 (62)
Smoking status								
Never	34 769 (51)	394 (53)	2850 (45)	594 (46)	194 872 (57)	767 (59)	3118 (45)	494 (51)
Previous	28 880 (42)	307 (42)	2537 (40)	575 (45)	109 867 (32)	450 (35)	2271 (33)	379 (39)
Current	4300 (6)	30 (4)	856 (14)	103 (8)	36 741 (11)	75 (6)	1450 (21)	100 (10)
Unknown	386 (1)	6 (1)	35 (1)	7 (1)	1134 (0)	2 (0)	48 (1)	5 (1)
eGFRcr, median (IQR), mL/min/1.73 m^2^	93 (83-97)	57 (54-59)	76 (69-85)	55 (51-58)	100 (90-106)	57 (54-59)	80 (71-91)	55 (51-58)
eGFRcys, median (IQR), mL/min/1.73 m^2^	80 (72-90)	69 (65-77)	55 (51-58)	50 (43-55)	93 (82-103)	75 (66-87)	56 (52-58)	49 (43-55)
eGFRcr-cys, median (IQR), mL/min/1.73 m^2^	89 (82-97)	66 (63-70)	66 (62-70)	53 (49-57)	99 (90-108)	68 (64-74)	67 (63-71)	53 (48-57)
uACR category								
<10 mg/g	59 155 (87)	640 (87)	5162 (82)	1004 (78)	311 560 (91)	1168 (90)	5803 (84)	746 (76)
10-29 mg/g	9180 (13)	94 (13)	1116 (18)	275 (22)	31 054 (9)	126 (10)	1084 (16)	232 (24)
Blood pressure, mean (SD), mm Hg								
Systolic	148 (20)	145 (20)	148 (20)	143 (19)	137 (19)	139 (20)	142 (19)	140 (20)
Diastolic	82 (10)	81 (10)	82 (11)	80 (11)	82 (11)	82 (11)	84 (11)	81 (11)
Cholesterol, mean (SD), mg/dL								
Total	221.6 (46.0)	219.5 (47.5)	213.0 (47.9)	203.9 (48.1)	222.1 (42.4)	222.0 (45.2)	216.4 (48.3)	208.4 (48.7)
HDL	57.2 (14.9)	57.4 (14.7)	51.1 (13.1)	51.2 (13.9)	56.5 (14.7)	57.5 (14.8)	(49.6) (13.2)	51.0 (13.5)
LDL	137.9 (34.7)	135.5 (35.9)	134.1 (36.3)	126.6 (36.7)	139.1 (32.5)	137.6 (34.4)	137.3 (36.4)	129.9 (36.7)
Diabetes	4187 (6)	48 (7)	663 (11)	173 (14)	11 960 (3)	62 (5)	776 (11)	159 (16)
Hypertension	22 439 (33)	280 (38)	2981 (47)	744 (58)	71 052 (21)	376 (29)	2933 (43)	573 (59)
Cholesterol-lowering medications	18 449 (27)	249 (34)	2104 (34)	583 (46)	37 238 (11)	242 (19)	1738 (25)	398 (41)
Blood pressure–lowering medications	9616 (14)	100 (14)	1458 (23)	336 (26)	28 743 (8)	156 (12)	1485 (22)	268 (27)

Abbreviations: CKD, chronic kidney disease; eGFRcr, estimated glomerular filtration rate based on serum creatinine level; eGFRcys, estimated glomerular filtration rate based on serum cystatin C level; eGFRcr-cys, estimated glomerular filtration rate based on serum creatinine and cystatin C levels; HDL, high-density lipoprotein; LDL, low-density lipoprotein; uACR, urine albumin to creatinine ratio.

SI conversion: To convert total, HDL, and LDL cholesterol to millimoles per liter, multiply by 0.0259.

^a^ Chronic kidney disease status was defined as no CKD (eGFRcr ≥60 mL/min/1.73 m^2^ and eGFRcys ≥60 mL/min/1.73 m^2^ [reference group]), Cr G3 (eGFRcr <60 mL/min/1.73 m^2^ and eGFRcys ≥60 mL/min/1.73 m^2^), Cys G3 (eGFRcr ≥60 and eGFRcys <60 mL/min/1.73 m^2^), and both G3 (eGFRcr <60 mL/min/1.73 m^2^ and eGFRcys <60 mL/min/1.73 m^2^).

### Discordance Between eGFRcr and eGFRcys

**Figure 1.**
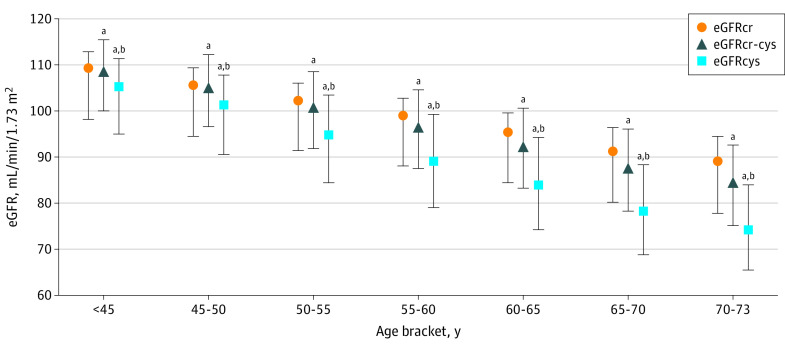
Median (IQR) Estimated Glomerular Filtration Rate Based on Serum Creatinine (eGFRcr), Estimated Glomerular Filtration Rate Based on Cystatin C (GFRcys), and eGFR Using Both Methods (eGFRcr-cys) Across 5-Year Age Brackets ^a^Significant difference noted with use of Wilcoxon signed rank test (*P* < .001) in median within each age bracket compared with eGFRcr. ^b^Significant difference noted with use of Wilcoxon signed rank test (*P* < .001) in median within each age bracket compared with eGFRcr-cys.

On assessment of cross-sectional baseline eGFR values, higher values of eGFRcr (>90 mL/min/1.73 m^2^) were generally associated with lower values of eGFRcys, especially in older age brackets (Figure 1; eFigure 1 in the Supplement). At lower values of eGFRcr (<60 mL/min/1.73 m^2^), eGFRcys values were generally higher in the younger age brackets.

This difference was more pronounced for eGFRcys than eGFRcr-cys: the median difference between eGFRcys and eGFRcr widened from 4 mL/min/1.73 m^2^ in the youngest group (age <45 years) to 14 mL/min/1.73 m^2^ in the oldest age category (70-73 years) (eTable 2 in the Supplement). The discrepancy was smaller between eGFRcys and 2009 eGFRcr (eTable 3, eFigure 2, and eFigure 3 in the Supplement).

Overall, the proportion of participants with discordant classifications of eGFR less than 60 mL/min/1.73 m^2^ was 9.2% of older participants and 2.4% in younger participants (eTable 4 in the Supplement). Among 2016 older participants with eGFRcr less than 60 mL/min/1.73 m^2^, 737 individuals (36.6%) had discordant eGFRcys values. The eGFRcr alone did not detect CKD in 6278 (8.2%) of older participants with eGFRcys less than 60 mL/min/1.73 m^2^. In sensitivity analyses, use of 2009 eGFRcr yielded similar proportions of discordance with eGFRcys as the main analyses (eTable 5 in the Supplement). The proportions with discordance between eGFRcr and eGFRcr-cys were much smaller in both older (2.4%) and younger (0.6%) participants (eTable 6 in the Supplement).

### Adverse Outcomes by CKD Category

**Figure 2.**
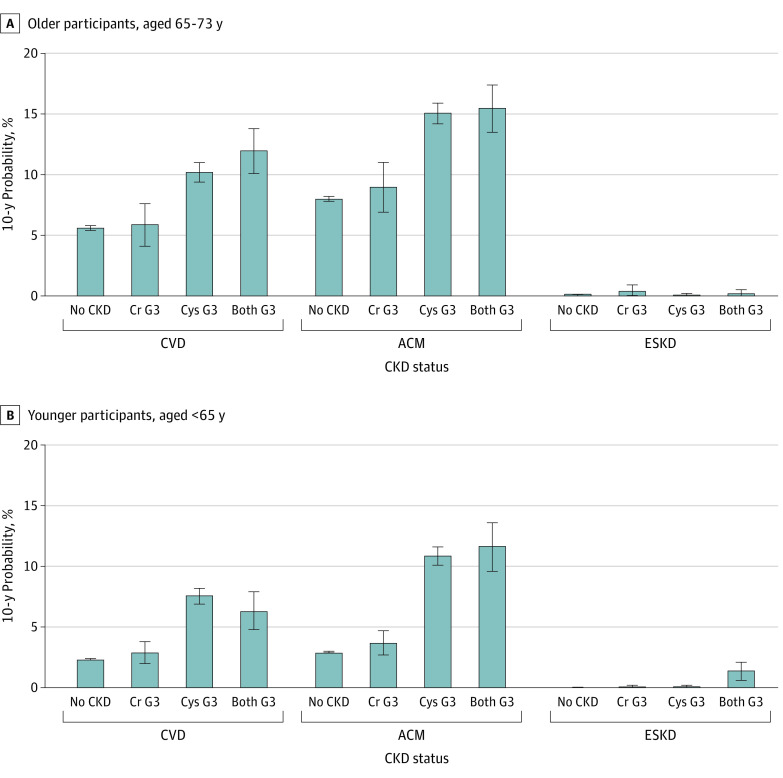
Ten-Year Probability of Outcomes of Interest According to Chronic Kidney Disease (CKD) Status Chronic kidney disease status in older (A) and younger (B) participants. Status was defined as no CKD (estimated glomerular filtration rate based on creatinine [eGFRcr] ≥60 mL/min/1.73 m^2^ and estimated glomerular filtration rate based on cystatin C [eGFRcys] ≥60 mL/min/1.73 m^2^ [reference group]), Cr G3 (eGFRcr <60 mL/min/1.73 m^2^ and eGFRcys ≥60 mL/min/1.73 m^2^), Cys G3 (eGFRcr ≥60 and eGFRcys <60 mL/min/1.73 m^2^), and both G3 (eGFRcr <60 mL/min/1.73 m^2^ and eGFRcys <60 mL/min/1.73 m^2^). ACM indicates all-cause mortality; Cr, creatinine; CVD, cardiovascular disease (fatal or nonfatal); Cys, cystatin C; and ESKD, end stage kidney disease (kidney failure).

The 10-year probability of kidney failure was very low in both age groups (older, 0.06%; younger, 0.04%). Across all CKD status groups, the 10-year probability of CVD and all-cause mortality outweighed the probability of kidney failure (Figure 2). In individuals with eGFRcys G3 alone or with both eGFRcr and eGFRcys CKD G3, the 10-year probability of CVD and all-cause mortality was nearly doubled in older adults, and more than doubled in younger adults, compared with those without CKD by either filtration marker. In contrast, persons with only eGFRcr G3 were not at substantially increased risk for any of these outcomes compared with those with no CKD. Similar patterns of results were observed when 2009 eGFRcr was tested for concordance with eGFRcys (eFigure 4 in the Supplement).

**Figure 3.**
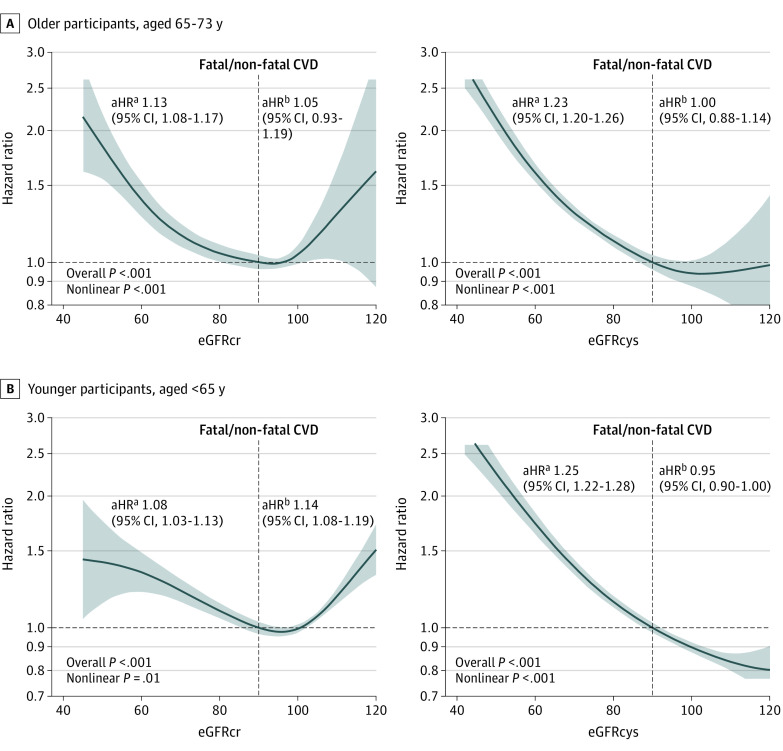
Adjusted Penalized Splines of the Hazards of Fatal and Nonfatal Cardiovascular Disease (CVD) Across the Spectrum of Estimated Glomerular Filtration Rate Based on Creatinine (eGFRcr) Level and Estimated Glomerular Filtration Rate Based on Cystatin C (eGFRcys) Level Adjusted hazard ratios (aHRs) in older (A) and younger (B) patients with fatal and nonfatal CVD after adjustment for atherosclerotic risk factors (age; sex; smoking status; systolic and diastolic blood pressure; total, high-density lipoprotein, and low-density lipoprotein cholesterol; history of diabetes or hypertension; and use of blood pressure– or cholesterol-lowering medications) and albuminuria. Nonlinear *P* values determined with the likelihood ratio test for addition of eGFR as a continuous variable to adjusted Cox proportional hazards regression model adjusted for atherosclerotic risk factors. Overall *P* values determined with the likelihood ratio test for addition of eGFR as a spline term to Cox proportional hazards regression model adjusted for atherosclerotic risk factors. Shaded areas indicate 95% CI. ^a^Adjusted HR per 10-mL/min/1.73 m^2^ decrease below eGFR 90 mL/min/1.73 m^2^. ^b^Adjusted HR per 10-mL/min/1.73 m^2^ increase above eGFR 90 mL/min/1.73 m^2^.

In both older and younger participants, there was an inflection point at approximately 90 mL/min/1.73 m^2^ for eGFRcr, below which there were increasing hazards of CVD (Figure 3); however, eGFRcr above this threshold was also associated with increased hazards of CVD. By comparison, eGFRcys showed a more linear associations with hazards of CVD.

In adjusted Cox proportional hazards regression models, individuals with evidence of CKD by both eGFRcr and eGFRcys, or by eGFRcys alone, had higher hazards of CVD and all-cause mortality compared with the reference group with neither eGFRcr nor eGFRcys evidence of CKD (eTable 7 in the Supplement). In both age groups, those with evidence of CKD G3 only with eGFRcr did not have higher hazards of CVD or death. Findings were very similar when 2009 eGFRcr was tested for concordance with eGFRcys (eTable 8 in the Supplement).

**Figure 4.**
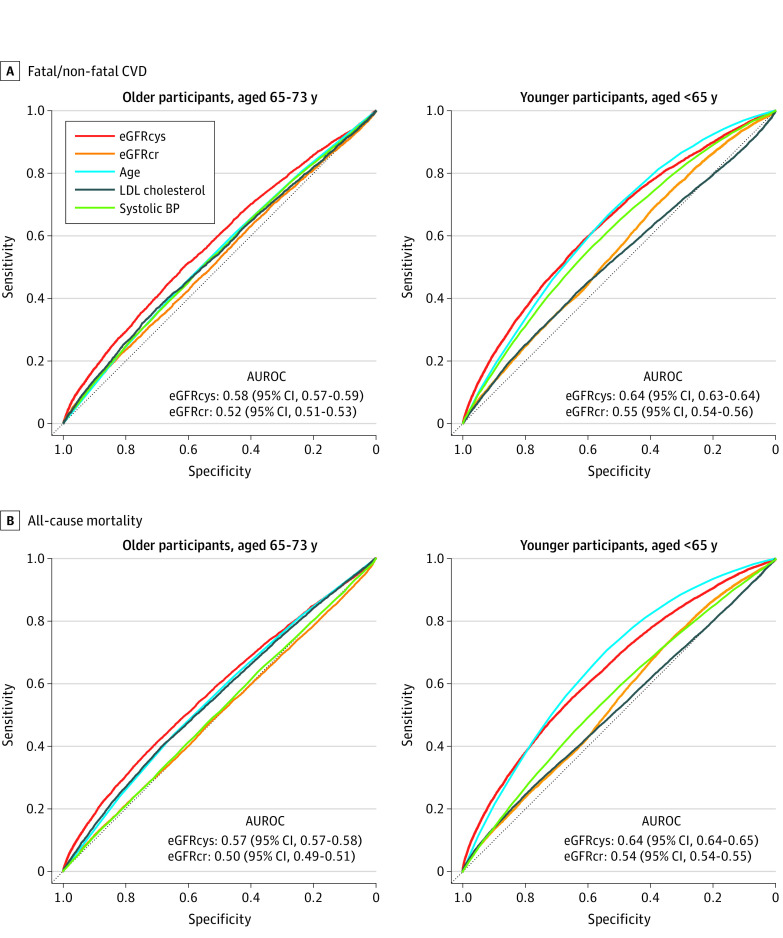
Area Under Receiver Operating Curves (AUROCs) for Individuals Demonstrating Discrimination of Estimated Glomerular Filtration Rate Based on Creatinine (eGFRcr) Level, Estimated Glomerular Filtration Rate Based on Cystatin C (eGFRcys) Level, and Other Traditional Risk Factors for the 2 Main Outcomes of Interest Fatal and nonfatal cardiovascular disease (CVD) and all-cause mortality shown for older (A) and younger (B) Patients. BP indicates blood pressure; LDL, low-density lipoprotein.

Compared with eGFRcr, area under receiver operating curves show that eGFRcys better discriminates risk of fatal and nonfatal CVD and all-cause mortality in both older and younger participants (Figure 4) and adds predictive discrimination to a base model of atherosclerotic risk factors for both CVD and all-cause mortality, while eGFRcr does not (eFigure 5 in the Supplement). Across a 7.5% 10-year risk threshold, case NRI improved by 0.7% (95% CI, 0.6%-0.8%) in older people and 0.7% (95% CI, 0.7%-0.8%) in younger people. This improvement in risk classification by eGFRcys in older people was greater than the contribution of diabetes (case NRI, 0.2%; 95% CI, 0.1%-0.3%) and similar to that of smoking history (case NRI, 0.4%; 95% CI, 0.2%-0.6%) and lipids (case NRI, 0.5%; 95% CI, 0.3%-0.7%) in this population. By comparison, the addition of eGFRcr did not improve risk classification of CVD in either older (0.0%) or younger (0.0%) people.

## Discussion

Among UK Biobank participants across a range of ages, the probabilities of CVD and all-cause mortality were substantially higher than the probability of kidney failure. Testing with eGFRcys identified a greater proportion of individuals with eGFR less than 60 mL/min/1.73 m^2^, particularly at older ages. In the absence of significant albuminuria, eGFRcys was both more sensitive and specific for high-risk CKD, better stratified individuals as having lower or higher risk of CVD and mortality compared with eGFRcr, and performed better than other accepted risk factors for CVD in the general population.

The eGFRcys has been shown to improve risk stratification across the threshold for CKD diagnosis.^20^ In a prospective cohort study of more than 26 000 participants in the US, eGFRcys substantially improved risk stratification for kidney failure and death compared with eGFRcr and albuminuria alone.^20^ In other previous studies, eGFRcys was associated with CVD risk across the spectrum of eGFR and improved risk prediction for CVD as much as established risk factors including lipids.^5^ By comparison, eGFRcr and eGFRcr-cys have an inconsistent association with these outcomes.^21,22,23^ To our knowledge, ours is the first study to assess the risks of CVD according to concordance testing using eGFRcys or eGFRcr-cys, and the first study to have adequate power to stratify by age. Compared with previous studies, our study has nearly half a million participants and almost triple the median follow-up.

Our findings have important implications for CKD guideline development. Age-adapted CKD thresholds were proposed in 2019,^19^ suggesting that the GFR threshold be lowered to 45 mL/min/1.73 m^2^ in individuals aged 65 years or older. The outcomes of this potential change were explored in a Canadian population study: Liu et al^24^ found that older individuals with eGFRcr 45 to 59 mL/min/1.73 m^2^ had similar risks of kidney failure and death as those without CKD. We found that the risks of CVD and mortality were not increased in those with eGFRcr less than 60 mL/min/1.73 m^2^ unless eGFRcys was also less than 60 mL/min.1.73 m^2^. However, 8% of older people in our population had eGFRcys less than 60 mL/min/1.73 m^2^ despite eGFRcr greater than or equal to 60 mL/min/1.73 m^2^: these individuals were at much higher risk of adverse outcomes. Our findings suggest that in the absence of eGFRcys testing, eGFRcr underestimates the broader risks of CVD and death associated with mild CKD.

We initially sought to validate the guideline-recommended approach^25^ to perform confirmatory testing for CKD using eGFRcys when eGFRcr is 45 to 59 mL/min/1.73 m^2^. For both older and younger adults, 1 case of CKD would be confirmed (and 1 case refuted) for every 2 eGFRcys tests performed. However, this approach would not capture individuals who have eGFRcys less than 60 mL/min/1.73 m^2^ but eGFRcr greater than or equal to 60 mL/min/1.73 m^2^. For a fixed number of tests, eGFRcys detected 4 times more individuals with eGFR less than 60 mL/min/1.73 m^2^ compared with eGFRcr (and more than twice as many individuals compared with eGFRcr_2009_). Increased CKD diagnosis by eGFRcys would enable enhanced eligibility for 3 important risk stratification and reduction strategies. First, assessment of CVD risk: current CVD risk estimation tools do not routinely account for eGFR, and the CKD patch (a multiplication factor for CVD risk to account for kidney function^26^) is based on eGFRcr. Our findings suggest that, in a general population cohort with relatively preserved kidney function, eGFRcr does not enhance risk estimation, while eGFRcys improves stratification with greater sensitivity and specificity than established, treatable cardiovascular risk factors (eg, lipid levels and blood pressure). Enhanced detection of high-risk individuals with eGFRcys, who may not otherwise qualify for CVD prevention therapies based on established CVD risk estimation tools, would enable earlier introduction of cost-effective CVD risk-reduction treatments, such as statins^27,28^ and sodium-glucose cotransporter-2 inhibitors (shown to reduce cardiovascular, kidney, and mortality risk in both diabetic and nondiabetic (albuminuric) CKD.^29^ Second, albuminuria testing,^1^ essential for estimation of kidney failure risk, is independently associated with CVD and all-cause mortality and is integral for treatment selection.^30^ Third, the kidney failure risk equation, developed and validated across multiple international cohorts, was used to estimate risk of kidney failure.^31,32,33^ The kidney failure risk equation is becoming an important gateway to nephrology care, requires albuminuria for calculation, and is only validated for use when eGFR is less than 60 mL/min/1.73 m^2^. There is no predictive advantage of using eGFRcr vs eGFRcys in the kidney failure risk equation to predict progression to kidney failure.^34^

Use of cystatin C as a filtration marker of kidney function has been criticized owing to the presence of non-GFR determinants including inflammation, obesity, diabetes, smoking, and thyroid disease.^35,36,37,38^ The use of eGFRcys may have a greater association with long-term outcomes because it is capturing aspects of cardiometabolic risk that are unrelated to kidney function. Conversely, there is evidence that non-GFR determinants affect eGFRcr (eg, muscle mass, physical activity, dietary meat/protein intake, and tubular secretion^3,39,40^). Serum creatinine levels consistently overestimate GFR and underestimate risk in individuals with lower muscle mass,^3^ which becomes more common with increasing age.^4^

Compared with creatinine, determination of cystatin C levels is not used widely in clinical practice: testing is available in fewer laboratories and cystatin C is more expensive.^41^ Prevention of CVD and kidney failure events may offset the additional costs of tests, although, to our knowledge, this has not been investigated. In younger individuals, who more often have a longer life expectancy and a greater lifetime risk of CVD and kidney failure, this strategy may be especially valuable. Moreover, measuring cystatin C levels allows the flexibility to report the combined equation (eGFRcr-cys), which reduces bias associated with non-GFR determinants of creatinine and cystatin C levels and is more valuable where accuracy is desired,^13,14^ eg, to optimize efficacy and safety in drug dosing.

### Strengths and Limitations

The strengths of this study are its large population size with detailed comorbidity and laboratory data and long follow-up. This study has limitations. First, we used single baseline measurements of creatinine, cystatin C, and albuminuria levels to define CKD. Second, the maximum age of participants in our cohort at baseline was 73 years. We cannot be certain that our observations can be generalized to older age groups. However, the discordance between eGFRcr and eGFRcys appears to augment with increasing age, suggesting that this outcome may be even more pronounced in older patients. Third, although eGFRcr and eGFRcys have discordant associations with long-term risk, participants in the UK Biobank did not have measured GFR levels; therefore, we cannot confirm that the discrepant level of risk associated with either measure is solely related to kidney function. Fourth, our stated conclusions about confirmatory testing with cystatin C apply in the setting of normal urine albumin concentration (<30 mg/g); we have not compared outcomes in participants who have eGFRcr 45 to 59 mL/min/1.73 m^2^ and albuminuria greater than 30 mg/g. Fifth, although we used the new equations for eGFR that do not include a race coefficient,^13^ the UK Biobank predominantly comprises White participants, limiting generalizability to other racial groups. Sixth, the CKD-EPI 2021 equation might introduce some level of bias in older individuals of European ancestry, in whom it has been shown to overestimate GFR, and therefore discordance with eGFRcys, compared with 2009 CKD-EPI.^42^ Seventh, UK Biobank is a volunteer cohort with a lower prevalence of chronic diseases, including CKD, CVD, diabetes, and hypertension, compared with the general population.^43^ There were small numbers of participants with advanced CKD, and a very small number of these progressed to kidney failure. The absolute risk of other adverse clinical events in the UK Biobank may be lower than in unselected populations. However, the relative risks should be generalizable to the UK population^44^ and those with eGFR less than 60 mL/min/1.73 m^2^ based on eGFRcys are almost certainly to be at higher risk of CVD and death. We do not report adjusted hazards of kidney failure owing to the extremely low incidence of this outcome among persons with eGFRcr greater than or equal to 45 mL/min/1.73 m^2^, even with a cohort of 428 402 individuals.

## Conclusions

In this study, categorization of CKD by eGFRcr alone included a large proportion of low-risk individuals and failed to capture a substantial proportion of individuals at higher risk for CVD and mortality. Testing using eGFRcys is more sensitive than eGFRcr and improves the specificity of diagnosis of high-risk CKD, where there are substantially higher risks of CVD and death than of kidney failure. These findings suggest that, in the absence of cystatin C testing, eGFRcr inadequately detects the broader risks associated with mild CKD.

## Notes

Supplement. **eMethods 1.** UK Biobank Participants **eMethods 2.** UK Biobank Algorithm for Determination of Kidney Failure Outcome **eMethods 3.** Method of Ascertainment of Area Under Receiving Operating Curve (AUROC) for Incremental Discrimination of eGFRcr and eGFRcys for CVD and Mortality
eReferences
**eTable 1.** Baseline Characteristics of Participants by CKD Status in Older (Age 65-73 Years) and Younger (Age <65 Years) Participants **eTable 2.** Cross-sectional Associations of 5-Year Age Increments With Kidney Function as Estimated by eGFRcr, eGFRcys and eGFRcr-cys **eTable 3.** Cross-sectional Associations of 5-Year Age Increments With Kidney Function as Estimated by 2009 eGFRcr, eGFRcys, and 2009 eGFRcr-cys **eTable 4.** Concordance of eGFRcr and eGFRcys in Older and Younger Participants **eTable 5.** Concordance of 2009 eGFRcr and eGFRcys in Older and Younger Participants **eTable 6.** Concordance of eGFRcr and eGFRcr-cys in Older and Younger Participants **eTable 7.** Cox Proportional Hazards of Fatal/Nonfatal Cardiovascular Disease and All-cause Mortality: Concordance Testing Between eGFRcr and eGFRcys **eTable 8.** Cox Proportional Hazards of Fatal/Nonfatal Cardiovascular Disease and All-cause Mortality: Concordance Testing Between 2009 eGFRcr and eGFRcys **eFigure 1.** Scatter Plot of eGFRcys Against eGFRcr and Locally Weighted Linear Regression (LOESS) Trend Lines Plotted for Each Age Bracket **eFigure 2.** Plot of Median (IQR) 2009 eGFRcr2009, eGFRcys, and 2009 eGFRcr-cys Across 5-Year Age Brackets **eFigure 3.** Scatter Plot of eGFRcys Against 2009 eGFRcr and Locally Weighted Linear Regression (LOESS) Trend Lines Plotted for Each Age Bracket **eFigure 4.** Bar Graphs Showing the 10-Year Probability of Outcomes of Interest (and 95% CIs) According to CKD Status **eFigure 5.** Receiving Operating Curves for Incremental Discrimination of eGFRcr and eGFRcys for CVD and Mortality

## References

[1] Kidney Disease Working Group. Kidney Disease: Improving Global Outcomes (KDIGO) 2012 clinical practice guideline for the evaluation and management of chronic kidney disease. Kidney Int Suppl. 2013;3(1):1-150. 10.1038/ki.2013.243 23989362

[2] Schaeffner ES, Ebert N, Kuhlmann MK, . Age and the course of GFR in persons aged 70 and above. Clin J Am Soc Nephrol. 2022;CJN.16631221. doi:10.2215/CJN.16631221 35850785 PMC9435992

[3] Nankivell BJ, Nankivell LFJ, Elder GJ, Gruenewald SM. How unmeasured muscle mass affects estimated GFR and diagnostic inaccuracy. EClinicalMedicine. 2020;29-30:100662. doi:10.1016/j.eclinm.2020.100662 33437955 PMC7788434

[4] Suetta C, Haddock B, Alcazar J, . The Copenhagen Sarcopenia Study: lean mass, strength, power, and physical function in a Danish cohort aged 20-93 years. J Cachexia Sarcopenia Muscle. 2019;10(6):1316-1329. doi:10.1002/jcsm.12477 31419087 PMC6903448

[5] Lees JS, Welsh CE, Celis-Morales CA, . Glomerular filtration rate by differing measures, albuminuria and prediction of cardiovascular disease, mortality and end-stage kidney disease. Nat Med. 2019;25(11):1753-1760. doi:10.1038/s41591-019-0627-8 31700174 PMC6858876

[6] Shlipak MG, Matsushita K, Ärnlöv J, ; CKD Prognosis Consortium. Cystatin C versus creatinine in determining risk based on kidney function. N Engl J Med. 2013;369(10):932-943. doi:10.1056/NEJMoa1214234 24004120 PMC3993094

[7] Lasserson DS, Shine B, O’Callaghan CA, James T. Requirement for cystatin C testing in chronic kidney disease: a retrospective population-based study. Br J Gen Pract. 2017;67(663):e732-e735. doi:10.3399/bjgp17X692585 28893765 PMC5604838

[8] Allen N, Sudlow C, Downey P, . UK Biobank: current status and what it means for epidemiology. Health Policy Technol. 2012;1:123-126. doi:10.1016/j.hlpt.2012.07.003

[9] UK Biobank. Protocol for a large-scale prospective epidemiological resource. March 21, 2007. Accessed September 10, 2022. https://www.ukbiobank.ac.uk/media/gnkeyh2q/study-rationale.pdf

[10] Elliott P, Peakman TC; UK Biobank. The UK Biobank sample handling and storage protocol for the collection, processing and archiving of human blood and urine. Int J Epidemiol. 2008;37(2):234-244. doi:10.1093/ije/dym276 18381398

[11] UK Biobank Showcase. Blood sample collection, processing and transport. 2011. Accessed April 17, 2019. https://biobank.ctsu.ox.ac.uk/showcase/docs/Bloodsample.pdf

[12] UK Biobank Showcase. Biospeciments manual: collection of biological samples, processing and storage. 2011. Accessed April 17, 2019. https://biobank.ctsu.ox.ac.uk/crystal/docs/BioSampleProc.pdf

[13] Inker LA, Eneanya ND, Coresh J, ; Chronic Kidney Disease Epidemiology Collaboration. New creatinine- and cystatin C–based equations to estimate GFR without race. N Engl J Med. 2021;385(19):1737-1749. doi:10.1056/NEJMoa2102953 34554658 PMC8822996

[14] Inker LA, Schmid CH, Tighiouart H, ; CKD-EPI Investigators. Estimating glomerular filtration rate from serum creatinine and cystatin C. N Engl J Med. 2012;367(1):20-29. doi:10.1056/NEJMoa1114248 22762315 PMC4398023

[15] Hsu CY, Yang W, Parikh RV, ; CRIC Study Investigators. Race, genetic ancestry, and estimating kidney function in CKD. N Engl J Med. 2021;385(19):1750-1760. doi:10.1056/NEJMoa2103753 34554660 PMC8994696

[16] Goff DC Jr, Lloyd-Jones DM, Bennett G, ; American College of Cardiology/American Heart Association Task Force on Practice Guidelines. ACC/AHA guideline on the assessment of cardiovascular risk: a report of the American College of Cardiology/American Heart Association task force on practice guidelines. Circulation. 2014;129(25)(suppl 2):S49-S73. 24222018 10.1161/01.cir.0000437741.48606.98

[17] UK Biobank. Algorithmically-defined health outcomes. January 2022. Accessed September 24, 2021. https://biobank.ndph.ox.ac.uk/showcase/showcase/docs/alg_outcome_main.pdf

[18] Bush K, Nolan J, Zhang Q, Herrington W, Sudlow C; UK Biobank Outcome Adjudication Group. Definitions of end stage renal disease for UK Biobank phase 1 outcomes adjudication. January 2022. Accessed September 10, 2022. https://biobank.ndph.ox.ac.uk/ukb/ukb/docs/alg_outcome_esrd.pdf

[19] Delanaye P, Jager KJ, Bökenkamp A, . CKD: a call for an age-adapted definition. J Am Soc Nephrol. 2019;30(10):1785-1805. doi:10.1681/ASN.2019030238 31506289 PMC6779354

[20] Peralta CA, Shlipak MG, Judd S, . Detection of chronic kidney disease with creatinine, cystatin C, and urine albumin-to-creatinine ratio and association with progression to end-stage renal disease and mortality. JAMA. 2011;305(15):1545-1552. doi:10.1001/jama.2011.468 21482744 PMC3697771

[21] Hallan SI, Matsushita K, Sang Y, ; Chronic Kidney Disease Prognosis Consortium. Age and association of kidney measures with mortality and end-stage renal disease. JAMA. 2012;308(22):2349-2360. doi:10.1001/jama.2012.16817 23111824 PMC3936348

[22] Matsushita K, van der Velde M, Astor BC, ; Chronic Kidney Disease Prognosis Consortium. Association of estimated glomerular filtration rate and albuminuria with all-cause and cardiovascular mortality in general population cohorts: a collaborative meta-analysis. Lancet. 2010;375(9731):2073-2081. doi:10.1016/S0140-6736(10)60674-5 20483451 PMC3993088

[23] Kühn A, van der Giet M, Kuhlmann MK, . Kidney function as risk factor and predictor of cardiovascular outcomes and mortality among older adults. Am J Kidney Dis. 2021;77(3):386-396.e1. doi:10.1053/j.ajkd.2020.09.015 33197533

[24] Liu P, Quinn RR, Lam NN, . Accounting for age in the definition of chronic kidney disease. JAMA Intern Med. 2021;181(10):1359-1366. doi:10.1001/jamainternmed.2021.4813 34459844 PMC8406213

[25] Kidney International Supplements. Definition and classification of CKD. January 2013. Accessed September 10, 2022. https://kdigo.org/wp-content/uploads/2017/02/KDIGO_2012_CKD_GL.pdf 10.1038/kisup.2012.64 PMC4089693 25018975

[26] Matsushita K, Jassal SK, Sang Y, . Incorporating kidney disease measures into cardiovascular risk prediction: development and validation in 9 million adults from 72 datasets. EClinicalMedicine. 2020;27:100552. doi:10.1016/j.eclinm.2020.100552 33150324 PMC7599294

[27] Herrington WG, Emberson J, Mihaylova B, ; Cholesterol Treatment Trialists’ (CTT) Collaboration. Impact of renal function on the effects of LDL cholesterol lowering with statin-based regimens: a meta-analysis of individual participant data from 28 randomised trials. Lancet Diabetes Endocrinol. 2016;4(10):829-839. doi:10.1016/S2213-8587(16)30156-5 27477773

[28] Kohli-Lynch CN, Lewsey J, Boyd KA, . Beyond ten-year risk: a cost-effectiveness analysis of statins for the primary prevention of cardiovascular disease. Circulation. 2022;145(17):1312-1323. doi:10.1161/CIRCULATIONAHA.121.057631 35249370 PMC9022692

[29] Vogt L, Stefansson B, Correa-Rotter R, . Dapagliflozin in patients with chronic kidney disease. N Engl J Med. 2021;384(4):388-389. doi:10.1056/NEJMc2032809 33503360

[30] Kang M, Kwon S, Lee J, . Albuminuria within the normal range can predict all-cause mortality and cardiovascular mortality. Kidney360. 2021;3(1):74-82. doi:10.34067/KID.0003912021 35368577 PMC8967601

[31] Major RW, Shepherd D, Medcalf JF, Xu G, Gray LJ, Brunskill NJ. The Kidney Failure Risk Equation for prediction of end stage renal disease in UK primary care: an external validation and clinical impact projection cohort study. PLoS Med. 2019;16(11):e1002955. doi:10.1371/journal.pmed.1002955 31693662 PMC6834237

[32] Tangri N, Grams ME, Levey AS, ; CKD Prognosis Consortium. Multinational assessment of accuracy of equations for predicting risk of kidney failure: a meta-analysis. JAMA. 2016;315(2):164-174. doi:10.1001/jama.2015.18202 26757465 PMC4752167

[33] Whitlock RH, Chartier M, Komenda P, . Validation of the Kidney Failure Risk Equation in Manitoba. Can J Kidney Health Dis. 2017;4:2054358117705372. doi:10.1177/2054358117705372 28491341 PMC5406122

[34] Bundy JD, Mills KT, Anderson AH, Yang W, Chen J, He J; CRIC Study Investigators. Prediction of end-stage kidney disease using estimated glomerular filtration rate with and without race: a prospective cohort study. Ann Intern Med. 2022;175(3):305-313. doi:10.7326/M21-2928 35007146 PMC9083829

[35] Panaich S, Veeranna V, Zalawadiya S, Kottam A, Afonso L. Association of cystatin C with measures of obesity and its impact on cardiovascular events among healthy US adults. J Am Coll Cardiol. 2013;61:E1420. doi:10.1016/S0735-1097(13)61420-5 25118891

[36] Goede DL, Wiesli P, Brändle M, . Effects of thyroxine replacement on serum creatinine and cystatin C in patients with primary and central hypothyroidism. Swiss Med Wkly. 2009;139(23-24):339-344. 19529992 10.4414/smw.2009.12654

[37] Rule AD, Bailey KR, Lieske JC, Peyser PA, Turner ST. Estimating the glomerular filtration rate from serum creatinine is better than from cystatin C for evaluating risk factors associated with chronic kidney disease. Kidney Int. 2013;83(6):1169-1176. doi:10.1038/ki.2013.7 23423253 PMC3661736

[38] Anderson AH, Yang W, Hsu C-Y, ; CRIC Study Investigators. Estimating GFR among participants in the Chronic Renal Insufficiency Cohort (CRIC) Study. Am J Kidney Dis. 2012;60(2):250-261. doi:10.1053/j.ajkd.2012.04.012 22658574 PMC3565578

[39] Patel SS, Molnar MZ, Tayek JA, . Serum creatinine as a marker of muscle mass in chronic kidney disease: results of a cross-sectional study and review of literature. J Cachexia Sarcopenia Muscle. 2013;4(1):19-29. doi:10.1007/s13539-012-0079-1 22777757 PMC3581614

[40] Baxmann AC, Ahmed MS, Marques NC, . Influence of muscle mass and physical activity on serum and urinary creatinine and serum cystatin C. Clin J Am Soc Nephrol. 2008;3(2):348-354. doi:10.2215/CJN.02870707 18235143 PMC2390952

[41] National Institute for Health and Care Excellence. Costing statement: chronic kidney disease: implementing the NICE guideline on chronic kidney disease. July 23, 2014. Accessed September 10, 2022. http://www.nice.org.uk/guidance/cg182/resources/cg182-chronic-kidney-disease-update-costing-statement2

[42] Delanaye P, Vidal-Petiot E, Björk J, . Performance of creatinine-based equations to estimate glomerular filtration rate in White and Black populations in Europe, Brazil, and Africa. Nephrol Dial Transplant. 2022;gfac241. doi:10.1093/ndt/gfac241 36002032

[43] Fry A, Littlejohns TJ, Sudlow C, . Comparison of sociodemographic and health-related characteristics of UK Biobank participants with those of the general population. Am J Epidemiol. 2017;186(9):1026-1034. doi:10.1093/aje/kwx246 28641372 PMC5860371

[44] Batty GD, Gale CR, Kivimäki M, Deary IJ, Bell S. Comparison of risk factor associations in UK Biobank against representative, general population based studies with conventional response rates: prospective cohort study and individual participant meta-analysis. BMJ. 2020;368:m131. doi:10.1136/bmj.m131 32051121 PMC7190071

