# Supplementary material for: Assessment of Cystatin C Level for Risk Stratification in Adults With Chronic Kidney Disease
Source: JAMA Netw Open. 2022 Oct 25;5(10):e2238300. doi: 10.1001/jamanetworkopen.2022.38300 (PMC9597396; doi:10.1001/jamanetworkopen.2022.38300)
Supplement: Supplement. — eMethods 1. UK Biobank Participants eMethods 2. UK Biobank Algorithm for Determination of Kidney Failure Outcome eMethods 3. Method of Ascertainment of Area Under Receiving Operating Curve (AUROC) for Incremental Discrimination of eGFRcr and eGFRcys for CVD and Mortality eReferences eTable 1. Baseline Characteristics of Participants by CKD Status in Older (Age 65-73 Years) and Younger (Age <65 Years) Participants eTable 2. Cross-sectional Associations of 5-Year Age Increments With Kidney Function as Estimated by eGFRcr, eGFRcys and eGFRcr-cys eTable 3. Cross-sectional Associations of 5-Year Age Increments With Kidney Function as Estimated by 2009 eGFRcr, eGFRcys, and 2009 eGFRcr-cys eTable 4. Concordance of eGFRcr and eGFRcys in Older and Younger Participants eTable 5. Concordance of 2009 eGFRcr and eGFRcys in Older and Younger Participants eTable 6. Concordance of eGFRcr and eGFRcr-cys in Older and Younger Participants eTable 7. Cox Proportional Hazards of Fatal/Nonfatal Cardiovascular Disease and All-cause Mortality: Concordance Testing Between eGFRcr and eGFRcys eTable 8. Cox Proportional Hazards of Fatal/Nonfatal Cardiovascular Disease and All-cause Mortality: Concordance Testing Between 2009 eGFRcr and eGFRcys eFigure 1. Scatter Plot of eGFRcys Against eGFRcr and Locally Weighted Linear Regression (LOESS) Trend Lines Plotted for Each Age Bracket eFigure 2. Plot of Median (IQR) 2009 eGFRcr2009, eGFRcys, and 2009 eGFRcr-cys Across 5-Year Age Brackets eFigure 3. Scatter Plot of eGFRcys Against 2009 eGFRcr and Locally Weighted Linear Regression (LOESS) Trend Lines Plotted for Each Age Bracket eFigure 4. Bar Graphs Showing the 10-Year Probability of Outcomes of Interest (and 95% CIs) According to CKD Status eFigure 5. Receiving Operating Curves for Incremental Discrimination of eGFRcr and eGFRcys for CVD and Mortality [file jamanetwopen-e2238300-s001.pdf]

# Supplemental Online Content

Lees JS, Rutherford E, Stevens KI, et al. Assessment of cystatin C level for risk stratification in adults with chronic kidney disease. *JAMA Netw Open.* 2022;5(10):e2238300. doi:10.1001/jamanetworkopen.2022.38300

**eMethods 1.** UK Biobank Participants

**eMethods 2.** UK Biobank Algorithm for Determination of Kidney Failure Outcome

**eMethods 3.** Method of Ascertainment of Area Under Receiving Operating Curve (AUROC) for Incremental Discrimination of eGFRcr and eGFRcys for CVD and Mortality

**eReferences**

**eTable 1.** Baseline Characteristics of Participants by CKD Status in Older (Age 65-73 Years) and Younger (Age <65 Years) Participants

**eTable 2.** Cross-sectional Associations of 5-Year Age Increments With Kidney Function as Estimated by eGFRcr, eGFRcys and eGFRcr-cys

**eTable 3.** Cross-sectional Associations of 5-Year Age Increments With Kidney Function as Estimated by 2009 eGFRcr, eGFRcys, and 2009 eGFRcr-cys

**eTable 4.** Concordance of eGFRcr and eGFRcys in Older and Younger Participants

**eTable 5.** Concordance of 2009 eGFRcr and eGFRcys in Older and Younger Participants

**eTable 6.** Concordance of eGFRcr and eGFRcr-cys in Older and Younger Participants

**eTable 7.** Cox Proportional Hazards of Fatal/Nonfatal Cardiovascular Disease and All-cause Mortality: Concordance Testing Between eGFRcr and eGFRcys

**eTable 8.** Cox Proportional Hazards of Fatal/Nonfatal Cardiovascular Disease and All-cause Mortality: Concordance Testing Between 2009 eGFRcr and eGFRcys

**eFigure 1.** Scatter Plot of eGFRcys Against eGFRcr and Locally Weighted Linear Regression (LOESS) Trend Lines Plotted for Each Age Bracket

© 2022 Lees JS et al. *JAMA Network Open*.

**eFigure 2.** Plot of Median (IQR) 2009 eGFRcr2009, eGFRcys, and 2009 eGFRcr-cys Across 5-Year Age Brackets

**eFigure 3.** Scatter Plot of eGFRcys Against 2009 eGFRcr and Locally Weighted Linear Regression (LOESS) Trend Lines Plotted for Each Age Bracket

**eFigure 4.** Bar Graphs Showing the 10-Year Probability of Outcomes of Interest (and 95% CIs) According to CKD Status

**eFigure 5.** Receiving Operating Curves for Incremental Discrimination of eGFRcr and eGFRcys for CVD and Mortality

This supplemental material has been provided by the authors to give readers additional information about their work.

© 2022 Lees JS et al. *JAMA Network Open*.

# eMethods 1

The UK Biobank invited participation by post from adults (target age 40-69 years) who were registered with the National Health Service between 2006 and 2010, and who lived within reasonable travelling distance of an assessment centre in the United Kingdom. The response rate to invitation was 5.5%[1].

The procedure of biochemical sampling for UK Biobank has been described previously[2–4]. Single baseline samples of blood serum and spot urine were collected from each participant and analysed at a central laboratory. Serum and urine creatinine were measured using an enzymatic (creatinase), IDMS-traceable, method on Beckman Coulter AU5400 instrument (coefficient of variation (CV) <2.8% over 3 levels of control)[5]. Serum cystatin C was measured by latex enhanced immunoturbidimetric method on a Siemens ADVIA 1800 instrument (CV <1.4% over 2 levels of control)[5]. Urine microalbumin was measured by immunoturbidimetric method using reagents and calibrators sourced from Randox Bioscience UK (CV <2.1% over 2 levels of control)[6].

# eMethods 2

UK Biobank algorithm for determination of kidney failure outcome

As follow-up biochemical data were not routinely available, kidney failure was defined from hospital admission data according to a pre-specified algorithm, using ICD10 and OPCS4 codes to identify participants who required maintenance kidney replacement therapy (KRT)[7]. Participants with a kidney transplant or undergoing peritoneal dialysis were assumed to be receiving maintenance KRT for kidney failure. For participants who received haemodialysis, this was assumed to be indicative of kidney failure only if there was an associated indicator of CKD stage 5 prior to KRT, or within 365 days of KRT[7].

## eMethods 3

Method of ascertainment of area under receiving operating curve (AUROC) for incremental discrimination of eGFRcr and eGFRcys for CVD and mortality.

Within older (age 65-73 years; n=76,629) and younger (age <65 years; n=351,773) age groups, participants were divided into training (80%) and test (20%) datasets. Logistic regression models were generated in the training datasets for both older and younger age groups for fatal/non-fatal cardiovascular disease (CVD) and all-cause mortality as follows:

- CVD risk factors: age, sex, smoking status, systolic and diastolic blood pressure, total, high-density lipoprotein (HDL) and low-density lipoprotein (LDL) cholesterol, history of diabetes or hypertension, use of blood pressure or cholesterol-lowering medications
- CVD risk factors + eGFRcr
- CVD risk factors + eGFRcys

In the test datasets, predictions of the likelihood of CVD and mortality were generated using the predict() function. Predicted values in training and test datasets were compared using AUROC (Supplementary eTable 8) and plotted in receiver operating curves (Supplementary eFigure 5).

## eReferences


1. Batty GD, Gale CR, Kivimäki M, Deary IJ, Bell S: Comparison of risk factor associations in UK Biobank against representative, general population based studies with conventional response rates: prospective cohort study and individual participant meta-analysis. *BMJ* 368: m131, 2020
2. Elliott P, Peakman TC: The UK Biobank sample handling and storage protocol for the collection, processing and archiving of human blood and urine. *Int. J. Epidemiol.* 37: 234–244, 2008
3. UK Biobank: UK Biobank Showcase. Blood Sample Collection, Processing and Transport. [Internet]. 2011 [cited 2019 Apr 17]
4. UK Biobank: UK Biobank showcase. Biospeciments manual: Collection of biological samples, processing and storage. [Internet]. 2011 [cited 2019 Apr 17]
5. UK Biobank's Enhancements Working Group: UK Biobank biomarker panel [Internet]. 1–4, 2014 [cited 2019 May 9]
6. UK Biobank: UK Biobank Showcase. Details of assays and quality control information for the urinary biomarker data. v1.0.
7. Bush K, Nolan J, Zhang Q, Herrington W, Sudlow C: Definitions of End Stage Renal Disease for UK Biobank Phase 1 Outcomes Adjudication Documentation prepared by : On behalf of UK Biobank Outcome Adjudication Group Definitions of End Stage Renal Disease , UK Biobank phase 1 outcomes adjudication. 2017


© 2022 Lees JS et al. *JAMA Network Open*. 

# eTable 1

Baseline characteristics of participants by CKD status in older (age 65-73 years) and younger (age <65 years) participants: concordance testing between eGFRcr and eGFRcr-cys

| | Older participants (65-73 years) | | | | Younger participants (< 65 years) | | | |
|---|---|---|---|---|---|---|---|---|
| **Baseline characteristics** | **Neither** | **Cr only** | **Cys C only** | **Both** | **Neither** | **Cr only** | **Cys C only** | **Both** |
| **N** | 73,571 | 795 | 1,042 | 1,221 | 348,498 | 1,274 | 1,003 | 998 |
| **Age (years): median [IQR]** | 67 [66, 68] | 67 [66, 68] | 67 [66, 69] | 67 [66, 69] | 55 [48, 60] | 60 [55, 62] | 61 [57, 63] | 61 [58, 63] |
| **Male sex: n(%)** | 34858 (47.4) | 290 (36.5) | 445 (42.7) | 529 (43.3) | 153974 (44.2) | 391 (30.7) | 362 (36.1) | 380 (38.1) |
| **Smoking status: n(%)** | | | | | | | | |
| **Never** | 37160 (50.5) | 431 (54.2) | 459 (44.0) | 557 (45.6) | 197523 (56.7) | 756 (59.3) | 467 (46.6) | 505 (50.6) |
| **Previous** | 30989 (42.1) | 327 (41.1) | 428 (41.1) | 555 (45.5) | 111805 (32.1) | 447 (35.1) | 333 (33.2) | 382 (38.3) |
| **Current** | 5008 (6.8) | 31 (3.9) | 148 (14.2) | 102 (8.4) | 37998 (10.9) | 69 (5.4) | 193 (19.2) | 106 (10.6) |
| **Unknown** | 414 (0.6) | 6 (0.8) | 7 (0.7) | 7 (0.6) | 1172 (0.3) | 2 (0.2) | 10 (1.0) | 5 (0.5) |
| **eGFRcr (ml/min/1.73m$^2$): median [IQR]** | 92 [81, 96] | 57 [55, 58] | 65 [62, 70] | 5 [50.9 57] | 99 [89, 105] | 57 [54, 58] | 66 [62, 71] | 54 [50, 57] |
| **eGFRcys (ml/min/1.73m$^2$): median [IQR]** | 78.8 [69.7, 88.7] | 68.6 [63.2, 75.7] | 46.9 [43.1, 50.2] | 48.9 [43.2, 53.9] | 92.8 [81.5, 103.1] | 75.8 [66.5, 87.4] | 46.8 [42.8, 50.3] | 49.6 [43.0, 55.4] |
| **eGFRcr-cys (ml/min/1.73m$^2$): median [IQR]** | 88 [79, 96] | 65 [62, 69] | 57 [54, 58] | 52 [48, 56] | 99 [89, 107] | 68 [63, 74] | 56 [54, 58] | 52 [48, 56] |
| **uACR category:** | | | | | | | | |
| **<10mg/g** | 63,505 (86.3) | 694 (87.3) | 812 (77.9) | 953 (78.1) | 316,590 (90.8) | 1,151 (90.3) | 773 (77.1) | 763 (76.5) |
| **10-29mg/g** | 10,066 (13.7) | 101 (12.7) | 230 (22.1) | 268 (21.9) | 31,908 (9.2) | 123 (9.7) | 230 (22.9) | 235 (23.5) |
| **Systolic blood pressure (mmHg)** | 147.7 (19.5) | 144.4 (19.7) | 146.5 (19.7) | 143.6 (19.2) | 137.3 (18.8) | 139.0 (19.7) | 140.6 (19.3) | 139.3 (19.7) |
| **Diastolic blood pressure (mmHg)** | 82.2 (10.4) | 80.6 (10.3) | 81.2 (10.6) | 80.2 (11.4) | 82.1 (10.6) | 81.6 (10.8) | 82.6 (11.2) | 81.1 (11.1) |
| **Total cholesterol (mmol/L)** | 5.7 (1.2) | 5.7 (1.2) | 5.3 (1.3) | 5.3 (1.2) | 5.7 (1.1) | 5.8 (1.2) | 5.3 (1.2) | 5.4 (1.3) |
| **HDL cholesterol (mmol/L)** | 1.5 (0.4) | 1.5 (0.4) | 1.3 (0.4) | 1.3 (0.4) | 1.5 (0.4) | 1.5 (0.4) | 1.3 (0.4) | 1.3 (0.4) |
| **LDL cholesterol (mmol/L)** | 3.6 (0.9) | 3.5 (0.9) | 3.3 (1.0) | 3.3 (1.0) | 3.6 (0.8) | 3.6 (0.9) | 3.3 (0.9) | 3.4 (1.0) |
| **Diabetes: n(%)** | 4690 (6.4) | 53 (6.7) | 160 (15.4) | 168 (13.8) | 12561 (3.6) | 54 (4.2) | 175 (17.4) | 167 (16.7) |
| **Hypertension: n(%)** | 24829 (33.7) | 302 (38.0) | 591 (56.7) | 722 (59.1) | 73462 (21.1) | 384 (30.1) | 523 (52.1) | 565 (56.6) |
| **Cholesterol-lowering medications: n(%)** | 20118 (27.3) | 271 (34.1) | 435 (41.7) | 561 (45.9) | 38621 (11.1) | 237 (18.6) | 355 (35.4) | 403 (40.4) |
| **Blood pressure medications: (%)** | 10789 (14.7) | 110 (13.8) | 285 (27.4) | 326 (26.7) | 29979 (8.6) | 159 (12.5) | 249 (24.8) | 265 (26.6) |

Summary of baseline demographic and clinical characteristics by CKD status in older participants (65-73 years) and younger participants (<65 years). CKD status was defined as: "No CKD": eGFRcr >60 and eGFRcys >60 (reference group), "eGFRcr G3": eGFRcr <60 and eGFRcys >60, "eGFRcys G3": eGFRcr >60 and eGFRcys <60, "Both G3": eGFRcr <60 and eGFRcys <60. All eGFR units are in mL/min/1.73m$^2$. eGFRcr: estimated glomerular filtration rate based on serum creatinine; eGFRcys: estimated glomerular filtration rate based on serum cystatin C; eGFRcr-cys: estimated glomerular filtration rate based on serum creatinine and cystatin C; HDL: high density lipoprotein; LDL: low density lipoprotein.

# eTable 2

Cross-sectional associations of 5-year age increments with kidney function as estimated by eGFRcr, eGFRcys and eGFRcr-cys

| Age bracket (years) | | eGFRcr (mL/min/1.73m$^2$) | | eGFRcys (mL/min/1.73m$^2$) | | eGFRcr-cys (mL/min/1.73m$^2$) | |
|---|---|---|---|---|---|---|---|
| | **N** | **Median** | **IQR** | **Median** | **IQR** | **Median** | **IQR** |
| **<45 (Intercept)** | 46,170 | 109 | 98; 113 | 105 | 95; 111 | 108 | 100;115 |
| **45-50** | 58,409 | -4 | -4; -3 | -4 | -4; -4 | -3 | -3; -3 |
| **50-55** | 66,692 | -7 | -7; -7 | -10 | -10; -8 | -8 | -8; -7 |
| **55-60** | 78,242 | -10 | -10; -10 | -16 | -17; -12 | -12 | -13; -11 |
| **60-65** | 102,260 | -14 | -14; -13 | -21 | -21; -17 | -16 | -17; -15 |
| **65-70** | 74,766 | -18 | -18; -16 | -27 | -26; -23 | -21 | -22; -19 |
| **70-73** | 1,863 | -20 | -20; -18 | -31 | -29; -27 | -24 | -25; -23 |

Quantile regression to estimate median (IQR) change in eGFR with increasing age across age brackets.

## eTable 3

Cross-sectional associations of 5-year age increments with kidney function as estimated by $eGFRcr_{2009}$, eGFRcys and $eGFRcr\text{-}cys_{2009}$

| **Age bracket (years)** | | **$eGFRcr_{2009}$ (mL/min/1.73m$^2$)** | | **eGFRcys (mL/min/1.73m$^2$)** | | **$eGFRcr\text{-}cys_{2009}$ (mL/min/1.73m$^2$)** | |
|---|---|---|---|---|---|---|---|
| | **N** | **Median** | **IQR** | **Median** | **IQR** | **Median** | **IQR** |
| **<45 (Intercept)** | 46,170 | 105 | 98; 110 | 105 | 95; 111 | 104 | 96;111 |
| **45-50** | 58,409 | -4 | -4; -4 | -4 | -4; -4 | -3 | -4; -4 |
| **50-55** | 66,692 | -8 | -7; -8 | -10 | -10; -8 | -9 | -9; -8 |
| **55-60** | 78,242 | -11 | -11; -11 | -16 | -17; -12 | -13 | -13; -12 |
| **60-65** | 102,260 | -15 | -15; -15 | -21 | -21; -17 | -17 | -18; -16 |
| **65-70** | 74,766 | -19 | -19; -18 | -27 | -26; -23 | -22 | -22; -21 |
| **70-73** | 1,863 | -22 | -22; -20 | -31 | -29; -27 | -25 | -26; -24 |

Quantile regression to estimate median (IQR) change in eGFR with increasing age across age brackets.

# eTable 4

Concordance of eGFRcr and eGFRcys in older and younger participants

**Older (65-73 years)**

| | | **eGFRcys <60** | **eGFRcys ≥60** | |
|---|---|---|---|---|
| **eGFRcr** | **<60** | 1,279<br>1.7% | 737<br>1.0% | 2,016 |
| | **≥60** | 6,278<br>8.2% | 68,335<br>89.2% | 74,613 |
| | | 7,557 | 69,072 | 76,629 |

**Younger (<65 years)**

| | | **eGFRcys <60** | **eGFRcys ≥60** | |
|---|---|---|---|---|
| **eGFRcr** | **<60** | 978<br>0.3% | 1,294<br>0.4% | 2,272 |
| | **≥60** | 6,887<br>2.0% | 342,614<br>97.4% | 349,501 |
| | | 7,865 | 343,908 | 351,773 |

Concordance tables of eGFRcr and eGFRcys in older (65-73 years) and younger (<65 years) participants. eGFRcr: estimated glomerular filtration rate based on serum creatinine; eGFRcys: estimated glomerular filtration rate based on serum cystatin C. eGFR measurements are in mL/min/1.73m$^2$.

# eTable 5

Concordance between eGFRcr$_{2009}$ and eGFRcys in older and younger participants

**Older (65-73 years)**

| | | eGFRcys <60 | eGFRcys ≥60 | Total |
|---|---|---|---|---|
| **eGFRcr$_{2009}$** | **<60** | 1,871 | 1,468 | 3,339 |
| | | 2.4% | 1.9% | |
| | **≥60** | 5,686 | 67,604 | 73,290 |
| | | 7.4% | 88.2% | |
| | | 7,557 | 69,072 | 76,629 |

**Younger (<65 years)**

| | | eGFRcys <60 | eGFRcys ≥60 | Total |
|---|---|---|---|---|
| **eGFRcr$_{2009}$** | **<60** | 1,415 | 2,302 | 3,717 |
| | | 0.4% | 0.7% | |
| | **≥60** | 6,450 | 341,606 | 348,056 |
| | | 1.8% | 97.1% | |
| | | 7,865 | 343,908 | 351,773 |

Concordance tables of eGFRcr$_{2009}$ and eGFRccys in older (65-73 years) and younger (<65 years) participants. eGFRcr$_{2009}$: estimated glomerular filtration rate based on serum creatinine from CKD-EPI 2009, including race coefficients; eGFRcys: estimated glomerular filtration rate based on cystatin C. eGFR measurements are in mL/min/1.73m$^2$.

## eTable 6

Concordance between eGFRcr and eGFRcr-cys in older and younger participants

**Older (65-73 years)**

| | | eGFRcr-cys <60 | eGFRcr-cys ≥60 | |
|---|---|---|---|---|
| eGFRcr | <60 | 1,221<br>2% | 1,042<br>1% | 2,263 |
| | ≥60 | 795<br>1% | 73,571<br>96% | 74,366 |
| | | 2,016 | 74,613 | 76,629 |

**Younger (<65 years)**

| | | eGFRcr-cys <60 | eGFRcr-cys ≥60 | |
|---|---|---|---|---|
| eGFRcr | <60 | 998<br><1% | 1,274<br><1% | 2,272 |
| | ≥60 | 1,003<br><1% | 348,498<br>99% | 349,501 |
| | | 2,001 | 349,772 | 351,773 |

Concordance tables of eGFRcr and eGFRcr-cys in older (65-73 years) and younger (<65 years) participants. eGFRcr: estimated glomerular filtration rate based on serum creatinine; eGFRcr-cys: estimated glomerular filtration rate based on serum creatinine and cystatin C. eGFR measurements are in mL/min/1.73m$^2$.

© 2022 Lees JS et al. *JAMA Network Open*.

# eTable 7

Cox proportional hazards of fatal/non-fatal cardiovascular disease and all-cause mortality: concordance testing between eGFRcr and eGFRcys

**Older (65-73 years): 76,629 participants**

| CKD status | Fatal/non-fatal CVD | | | All-cause mortality | | |
|---|---|---|---|---|---|---|
| | N events | HR (95% CI) | P | N events | HR (95% CI) | P |
| **No CKD (n=68,335)** | 4,288 | 1 (Ref) | | 7,700 | 1 (Ref) | |
| **eGFRcr G3 (n=737)** | 47 | 1.11 (0.83-1.47) | 0.496 | 85 | 1.11 (0.89-1.37) | 0.350 |
| **eGFRcys G3 (n=6,728)** | 703 | 1.64 (1.51-1.78) | <0.001 | 1,284 | 1.69 (1.59-1.80) | <0.001 |
| **Both G3 (n= 1,279)** | 167 | 1.93 (1.65-2.26) | <0.001 | 266 | 1.71 (1.51-1.93) | <0.001 |

**Younger (< 65 years): 351,773 participants**

| CKD status | Fatal/non-fatal CVD | | | All-cause mortality | | |
|---|---|---|---|---|---|---|
| | N events | HR (95% CI) | P | N events | HR (95% CI) | P |
| **No CKD (n=342,614)** | 8,661 | 1 (Ref) | | 13,565 | 1 (Ref) | |
| **eGFRcr G3 (n= 1,294)** | 40 | 1.11 (0.81-1.51) | 0.530 | 65 | 1.05 (0.82-1.34) | 0.704 |
| **eGFRcys G3 (n=6,887)** | 559 | 1.86 (1.70-2.03) | <0.001 | 1,001 | 2.12 (1.98-2.26) | <0.001 |
| **Both G3 (n=978)** | 68 | 1.61 (1.27-2.05) | <0.001 | 145 | 2.13 (1.80-2.51) | <0.001 |

Cox proportional hazards models for fatal/non-fatal cardiovascular disease (the first of myocardial infarction, stroke or cardiovascular death) and all-cause mortality presented as hazard ratio (HR) with 95% confidence intervals. All models were adjusted for atherosclerotic risk factors (age, sex, smoking status, systolic and diastolic blood pressure, total, high-density lipoprotein (HDL) and low-density lipoprotein (LDL) cholesterol, history of diabetes or hypertension, and use of blood pressure or cholesterol-lowering medications) and albuminuria. Results are displayed for older participants (65-73 years; top) and younger participants (<65 years; bottom), with concordance testing between eGFRcr and eGFRcys. CKD status was defined as: "No CKD": eGFRcr ≥60 and eGFRcys ≥60 (reference group), "eGFRcr G3": eGFRcr <60 and eGFRcys ≥60, "eGFRcys G3": eGFRcr ≥60 and eGFRcys <60, "Both G3": eGFRcr <60 and eGFRcys <60. All eGFR units are in mL/min/1.73$m^2$.

# eTable 8

Cox proportional hazards of fatal/non-fatal cardiovascular disease and all-cause mortality: concordance testing between $eGFRcr_{2009}$ and eGFRcys

**Older (65-73 years): 76,629 participants**

| CKD status | Fatal/non-fatal CVD | | | All-cause mortality | | |
|---|---|---|---|---|---|---|
| | N events | HR (95% CI) | P | N events | HR (95% CI) | P |

| | | | | | | |
|---|---|---|---|---|---|---|
| **Neither (n=67,604)** | 4,239 | 1 (Ref) | | 7,617 | 1 (Ref) | |
| **Creat (n=1,468)** | 96 | 1.08 (0.88-1.32) | 0.460 | 168 | 1.05 (0.90-1.22) | 0.555 |
| **Cys C (n=5,686)** | 629 | 1.62 (1.49-1.77) | <0.001 | 1,172 | 1.70 (1.60-1.81) | <0.001 |
| **Both (n=1,871)** | 241 | 1.91 (1.67-2.18) | <0.001 | 378 | 1.67 (1.51-1.86) | <0.001 |
| **Younger (< 65 years): 351,773 participants** | | | | | | |
| **CKD status** | **Fatal/non-fatal CVD** | | | **All-cause mortality** | | |
| | **N events** | **HR (95% CI)** | **P** | **N events** | **HR (95% CI)** | **P** |
| **Neither (n=341,606)** | 8,626 | 1 (Ref) | | 13,515 | 1 (Ref) | |
| **Creat (n=2,302)** | 75 | 1.11 (0.89-1.40) | 0.347 | 115 | 1.00 (0.83-1.20) | 0.977 |
| **Cys C (6,450)** | 523 | 1.87 (1.70-2.04) | <0.001 | 946 | 2.15 (2.00-2.30) | <0.001 |
| **Both (n=1,415)** | 104 | 1.67 (1.37-2.02) | <0.001 | 200 | 1.99 (1.73-2.29) | <0.001 |

Cox proportional hazards models for fatal/non-fatal cardiovascular disease (the first of myocardial infarction, stroke or cardiovascular death) and all-cause mortality presented as hazard ratio (HR) with 95% confidence intervals. All models were adjusted for atherosclerotic risk factors (age, sex, smoking status, systolic and diastolic blood pressure, total, high-density lipoprotein (HDL) and low-density lipoprotein (LDL) cholesterol, history of diabetes or hypertension, and use of blood pressure or cholesterol-lowering medications) and albuminuria. Results are displayed for older participants (65-73 years; top) and younger participants (<65 years; bottom), with concordance testing between eGFRcr$_{2009}$ and eGFRcys. CKD status was defined as: "No CKD": eGFRcr$_{2009}$ $\geq$60 and eGFRcys $\geq$60 (reference group), "eGFRcr G3": eGFRcr$_{2009}$ <60 and eGFRcys $\geq$60, "eGFRcys G3": eGFRcr$_{2009}$ $\geq$60 and eGFRcys <60, "Both G3": eGFRcr$_{2009}$ <60 and eGFRcys <60. All eGFR units are in mL/min/1.73$m^2$.

eFigure 1

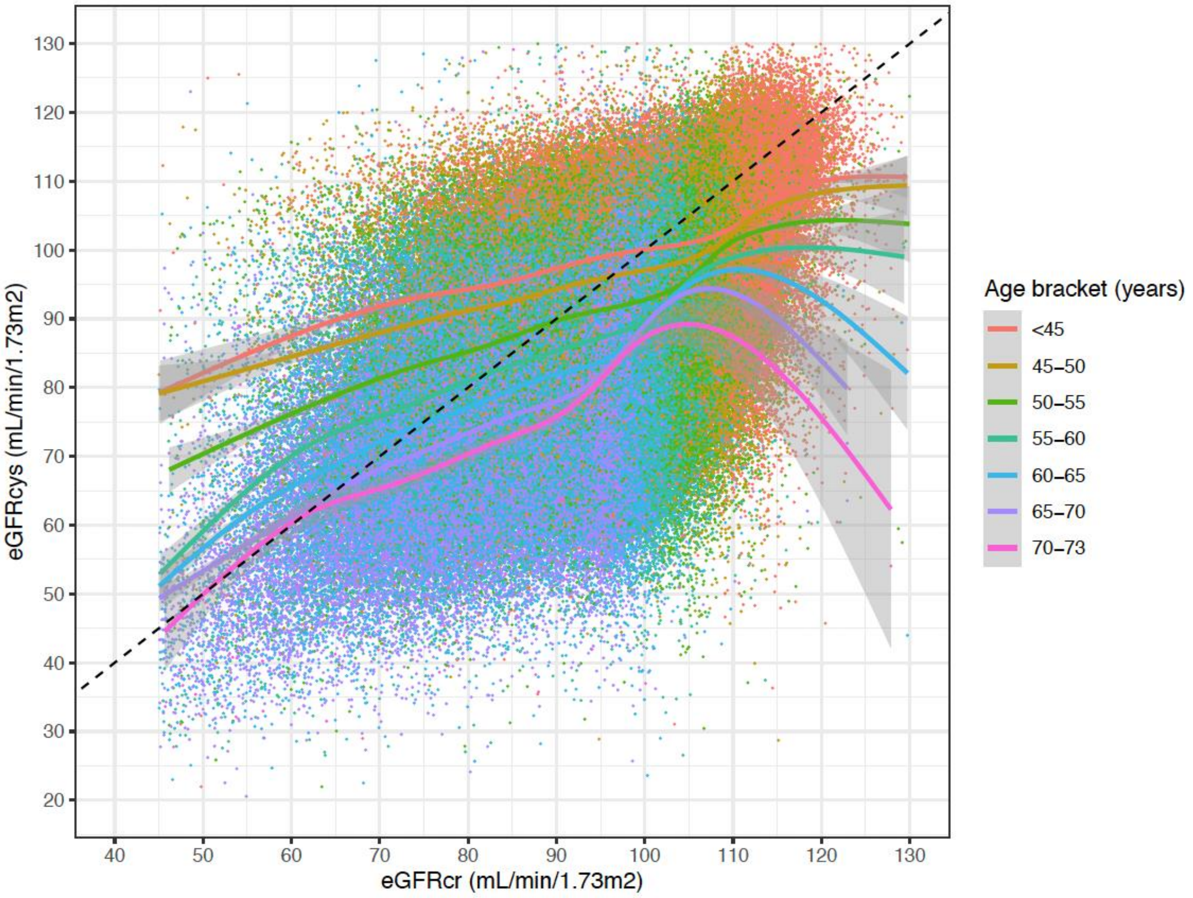


Scatter plot of eGFRcys against eGFRcr and locally-weighted linear regression (LOESS) trend lines plotted for each age bracket.

eFigure 2

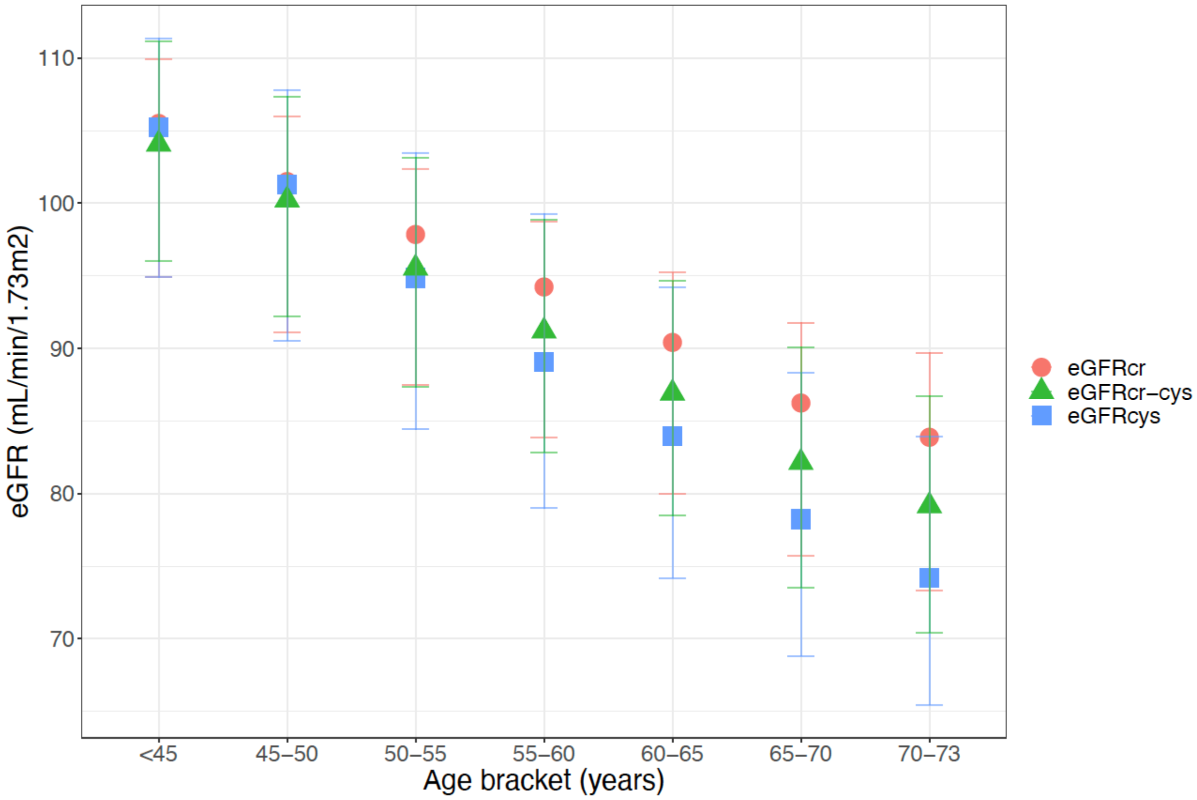


Plot of median (IQR) $eGFRcr_{2009}$, eGFRcys and $eGFRcr\text{-}cys_{2009}$ across 5-year age brackets.

© 2022 Lees JS et al. *JAMA Network Open*. 

## eFigure 3

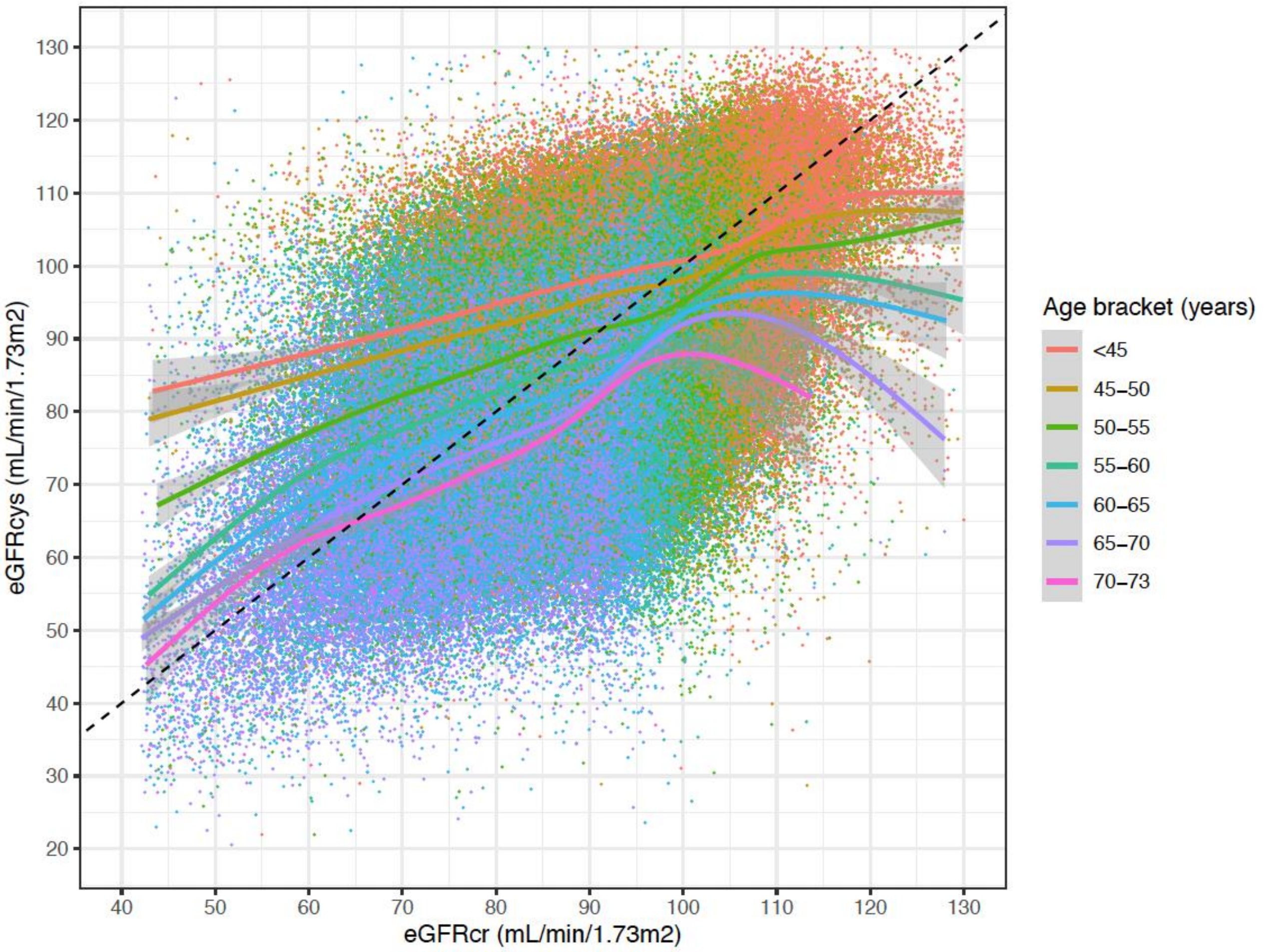


Scatter plot of eGFRcys against $eGFRcr_{2009}$ and locally-weighted linear regression (LOESS) trend lines plotted for each age bracket.

© 2022 Lees JS et al. *JAMA Network Open*. 

# eFigure 4

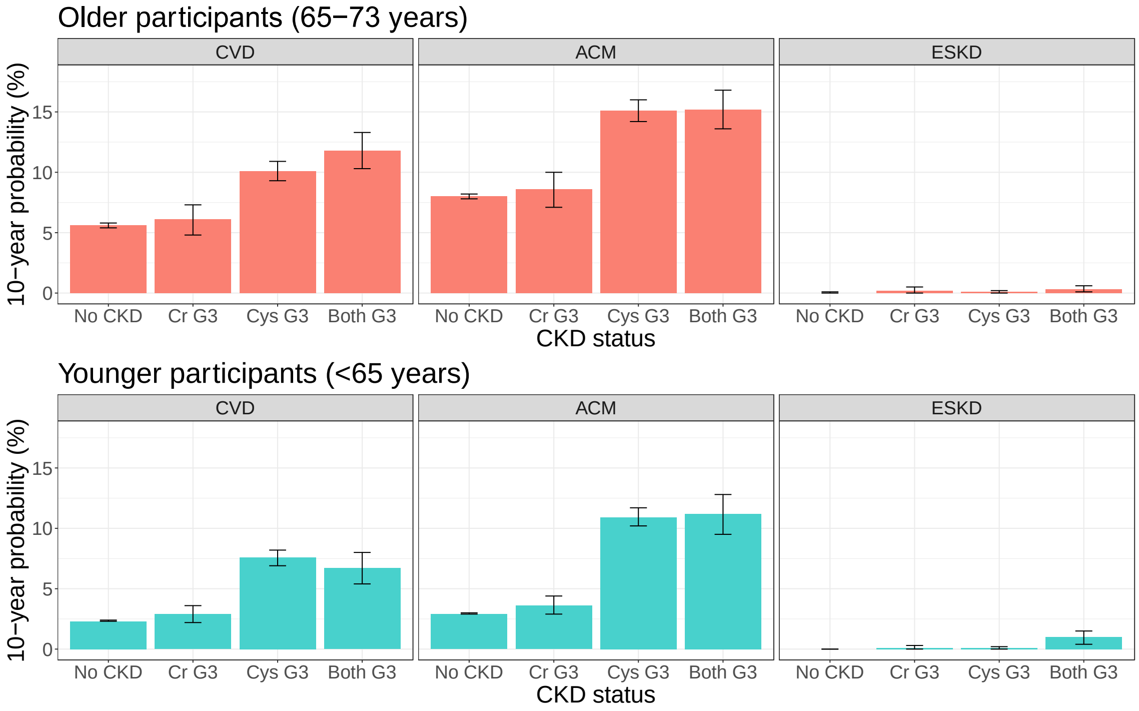


Bar graphs showing the 10-year probability of outcomes of interest (and 95% confidence intervals) according to CKD status. CKD status was defined as: “No CKD”: $eGFRcr_{2009} \geq 60$ and eGFRcys $\geq 60$ (reference group), “eGFRcr G3”: $eGFRcr_{2009}$ <60 and eGFRcys $\geq 60$, “eGFRcys G3”: $eGFRcr_{2009} \geq 60$ and eGFRcys <60, “Both G3”: $eGFRcr_{2009}$ <60 and eGFRcys <60. All eGFR units are in mL/min/1.73m$^2$. CVD: Fatal/non-fatal cardiovascular disease. ACM: all-cause mortality. ESKD: end-stage kidney disease.

## eFigure 5

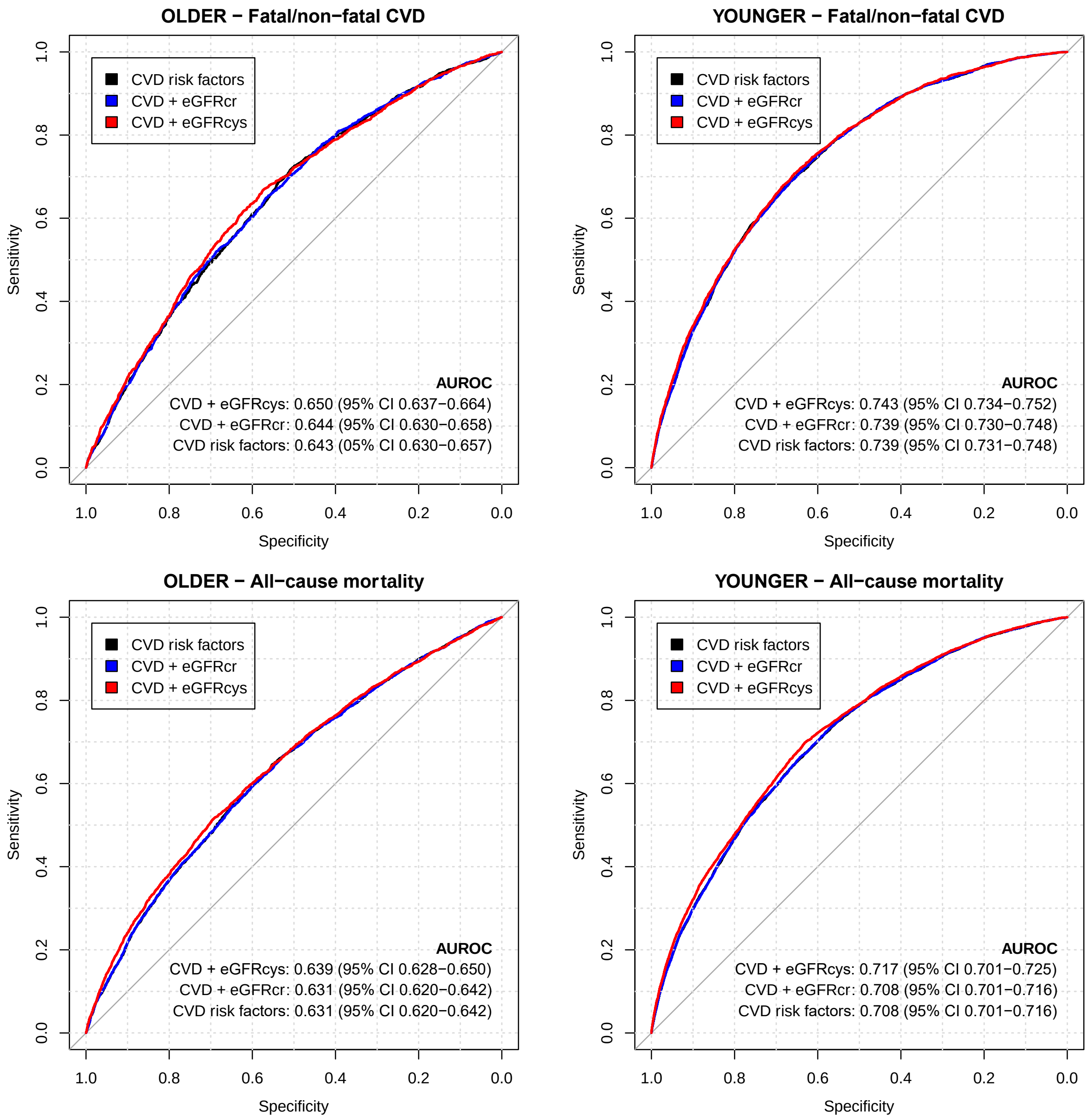


Receiving operating curves for incremental discrimination of eGFRcr and eGFRcys for CVD and mortality. CVD risk factors: cardiovascular disease risk factors (age, sex, smoking status, systolic and diastolic blood pressure, total, high-density lipoprotein (HDL) and low-density lipoprotein (LDL) cholesterol, history of diabetes or hypertension, and use of blood pressure or cholesterol-lowering medications). eGFRcr: estimated glomerular filtration rate based on serum creatinine. eGFRcys: estimated glomerular filtration rate based on cystatin C. AUROC: area under receiver operating curve.
